# Recognition of ear, nose and throat involvement in patients with ANCA-associated vasculitis: from in-depth interviews to insights for the future

**DOI:** 10.1007/s00405-026-10517-0

**Published:** 2026-08-07

**Authors:** Roline M. Krol, Jeroen Christiaans, Annelies Frima, Digna M.A. Kamalski, Julia Spierings, Marlies C. van der Goes

**Affiliations:** 1 Department of Clinical Immunology and Rheumatology, University Medical Centre Utrecht, Utrecht, the Netherlands; 2 Department of Clinical Immunology and Rheumatology, Amsterdam University Medical Centres, University of Amsterdam, Amsterdam, the Netherlands; 3 Department of Otorhinolaryngology, Meander Medical Centre, Amersfoort, the Netherlands; 4 Department of Otorhinolaryngology, University Medical Centre Utrecht, Utrecht, the Netherlands; 5 Department of Rheumatology, Meander Medical Centre, Amersfoort, the Netherlands

**Keywords:** Anti-Neutrophil Cytoplasmic Antibody-Associated Vasculitis, Otorhinolaryngologic Diseases, Systemic Vasculitis, Granulomatosis with Polyangiitis, Microscopic Polyangiitis, Eosinophilic Granulomatosis with Polyangiitis

## Abstract

**Purpose:**

Ear, nose, and throat (ENT) manifestations are common in antineutrophil cytoplasmic antibody–associated vasculitis (AAV). Although timely intervention is crucial, diagnosis and treatment are often delayed due to its rarity and nonspecific symptoms. Currently, no standardized tool exists to assess AAV activity in the ENT region.

This study aims to determine key indicators of active AAV in the ENT region, thereby supporting the development of a systematic examination method.

**Methods:**

A qualitative exploratory multicentre study was conducted. In-depth interviews with four otorhinolaryngologists were thematically analysed. Identified ENT symptoms and rhinoscopic findings associated with AAV were compiled, and statements regarding diagnosis and management were formulated. These items then formed the basis of an online survey presented to Dutch otorhinolaryngologists. Respondents rated items on a 0–10 agreement scale.

**Results:**

Of 29 initiated surveys, 25 (86.2%) were completed. Highest-rated symptoms supporting active AAV included nasal crusting (9.7/10), systemic symptoms (7.5/10), no effect of conventional therapy for chronic rhinosinusitis (7.6/10) and epistaxis (7.5/10). Most suspect rhinoscopic findings were nasal crusting (9.3/10), mucosal haemorrhaging (8.8/10) and septal perforation (8.6/10). Respondents strongly agreed that ENT involvement should be monitored by an otorhinolaryngologist (8.3/10), nasal lavage should precede rhinoscopy (7.6/10) and thorough nasal hygiene instructions should be provided (9.1/10).

**Conclusion:**

Key indicators of AAV activity include nasal crusting, lack of response to conventional treatment, systemic symptoms, nasal crusting, mucosal haemorrhaging and septal perforation. These data may facilitate the establishment of a standardized scoring system to optimize diagnostic accuracy and disease monitoring.

**Supplementary Information:**

The online version contains supplementary material available at https://doi.org/10.1007/s00405-026-10517-0.

## Introduction

Antineutrophilic cytoplasmic antibody – associated vasculitis (AAV) is a group of auto-immune disorders characterized by inflammation of the small blood vessels and the development of anti-neutrophilic cytoplasmic antibodies [1] AAV includes three different entities: granulomatosis with polyangiitis (GPA), microscopic polyangiitis (MPA), and eosinophilic granulomatosis with polyangiitis (EGPA) [2].

AAV has a broad clinical spectrum and can be life threatening in case of major organ involvement such as renal and pulmonary involvement [3]. Therefore, these organs are most known to be affected. However, a large group of patients mainly suffers from symptoms in the ear, nose and throat (ENT) region [4]. These symptoms may be the result of laryngeal, pharyngeal, otologic, orbital and sinonasal manifestations [4]. If left untreated, these manifestations can lead to irreversible damage. Up to 72% of patients with GPA have ENT involvement at diagnosis, in 58% sinonasal symptoms are present and 35% of patients suffer from otologic involvement [5]. Patients with EGPA have ENT manifestations in 48–96% at diagnosis, whereas ENT involvement in MPA is less common (20–30%) [6–8]. Despite the high prevalence of ENT involvement the diagnostic process, follow-up and treatment of ENT involvement should be improved. The diagnostic delay is often long in AAV patients with ENT involvement, due to the large variety of ENT symptoms and the lack of awareness for AAV in the ENT region [9, 10]. Furthermore, AAV can mimic the clinical presentation of other, more common diseases such as chronic rhinosinusitis or asthma [11]. In a Dutch study conducted by Dirikgil et al. the time to diagnosis was significantly longer in patients that were primarily assessed by an ENT-specialist (median 57 days versus 22 days in all patients with non-generalized disease) [10]. Currently there is no standardized scoring method to help differentiate between ENT involvement in AAV and other diseases that present with similar symptoms.

This proposes two main issues:

1. Suboptimal recognition of early-stage disease. If AAV presents with ENT manifestations only, it is often misdiagnosed. Delayed diagnosis also delays adequate treatment and can lead to irreversible damage.
2. Difficulty in follow-up due to the lack of standardized scoring methods for disease activity in the ENT region differentiating active vasculitis from persistent damage or infection, and limited experience of otorhinolaryngologists caused by the low incidence of AAV.

Therefore, this study aims to identify the principal factors for accurately detecting and evaluating disease activity within the ENT region in patients with AAV. The findings are intended to provide a foundation for the development of a standardized, methodical, and systematic examination protocol of the ENT region. This would enable otorhinolaryngologists to reliably quantify and score AAV-related disease activity, thereby improving diagnostic precision and facilitating more effective patient management.

## Materials and methods

### Design

This qualitative exploratory multicentre study was conducted between March 2022 and April 2024 in the Netherlands. In-depth interviews with otorhinolaryngologists were conducted and the results were thematically analysed. From the interviews, characteristics of active AAV and recommendations for management in clinical practice were extracted. The findings were then presented to other otorhinolaryngologists in the Netherlands. Otorhinolaryngologist were asked to score these characteristics and recommendations to assess the level of agreement.

The aim of this approach was to establish:

1. An overview of the principal characteristics of active AAV as seen during rhinoscopy.
2. Recommendations for appropriate follow-up and clinical management of patients with AAV in the ENT-region that could form the foundation for the development of a standardized scoring method for ENT-activity.

### In-depth interviews

#### Participants

We sought to include Dutch otorhinolaryngologists who had five or more years of experience with treating AAV. Otorhinolaryngologists with expertise in this field in both academic centres and regional hospitals were deemed eligible for interviewing. In close collaboration with the rhinology working group of the Dutch ENT society, seven experts with specific expertise were chosen and invited for in-depth interviews, all working in academic hospitals and/or vasculitis expertise centres. Three ENT-specialists declined the invitation due to scheduling issues. Four otorhinolaryngologists from three centres were interviewed.

#### Data collection and analyses

For the face-to-face, in-depth and semi-structured interviews, an interview guide with open-ended questions was designed (J.C.). (see appendix I. interview guide). A semi-structured approach was chosen to systematically work through formulated questions, but simultaneously leave room for further exploration of the given answers and for recording of additional statements. The aim of the questions in this interview was to understand how otorhinolaryngologists handle patients with AAV from diagnosis to follow-up. To achieve this objective, the questions were categorized into three domains: (1) diagnostic approaches for AAV in the ENT-region, (2) appropriate follow-up strategies, and (3) hygiene recommendations for the ENT-region.

The interviews were all conducted by one investigator (J.C.). After verbal informed consent was obtained, the audio of every interview was recorded. The interviews were then transcribed and the manuscripts were anonymized.

Interviews were analysed via a mostly deductive thematic analysis. Transcripts were analysed using the constant comparative technique [12, 13]. The data was independently coded and divided into themes by three of the investigators (J.C., M.G., A.F.). After coding, the data was sorted for clarification. For this process the QUAGOL guide for the analysis of qualitative research data was used [14]. Because the sample size consisted of four interviews, the use of coding software was deemed unnecessary. Saturation was assessed after each interview [14]. The consolidated criteria for report qualitative research (COREQ) were followed and are reported in appendix 7. via the COREQ checklist (COnsolidated crirteria for REporting Qualitative research) [15].

#### Extracted themes

Three main themes were extracted from the interviews: diagnosis of AAV in the ENT-region (both symptoms of active AAV and rhinoscopic findings), strategies for appropriate follow-up and at-home instructions for patients (Table 1).

**Table 1:** Themes extracted from in-depth interviews

Diagnosis of AAV in the ENT-region	Symptoms of active AAV
	Manifestations seen during rhinoscopy
	Differential diagnosis
Strategies for appropriate follow-up	Appointment frequency
	Cleaning of nasal cavity at every visit
	Both physical examination and patient history
	Hygiene advice for ENT-region
	Multidisciplinary regimen
Instructions for patients	No digital manipulation of nasal cavity
	High-flow nasal irrigation with saline
	Nasal ointment

### Survey

After analysing the interviews, coding and identifying the key themes, a list of the most important ENT symptoms and rhinoscopic manifestations of AAV was created. Next, statements on diagnosing AAV, follow-up and hygiene of the ENT region were formulated and included in an online survey that was created with QuestionPro software (QuestionPro Inc. Dallas, TX, USA.) The survey comprised of questions regarding the experience of otorhinolaryngologists with AAV and included statements regarding symptoms, findings during physical examination, differential diagnosis and follow-up. The level of agreement (on a 0–10 scale) on these statements was assessed. The survey was distributed via e-mail by the Dutch society of ENT specialists (De Vereniging voor Keel-Neus-Oorheelkunde en Heelkunde van het Hoofd-Halsgebied) amongst their members with experience in rhinology and treating patients with AAV (see appendix II survey). Results of the surveys were summarized.

## Results

A total of 36 otorhinolaryngologist were invited to complete the questionnaire. Twenty-nine questionnaires were started, 25 (86.2%) were completed. Three-quarters of the respondents were working in regional clinics. Years of experience as otorhinolaryngologist and number of patients treated with AAV specifically differed among respondents (Table 2).

**Table 2:** Baseline data respondents N= 29

	N (%)
Completed surveys	25 (86.2)
Current workplace, available for *N* = 29	
University hospital	6 (20.7)
Regional hospital	23 (79.3)
Years of experience as otorhinolaryngologist, available for *N* = 29	
0–5	8 (27.6)
5–10	9 (31.0)
>10	12 (41.4)
N of AAV patients treated by the otorhinolaryngologist, available for *N* = 29	
0–10	8 (27.6)
10–20	10 (34.5)
>20	11 (37.9)

### Presenting symptoms

**Fig. 1:**
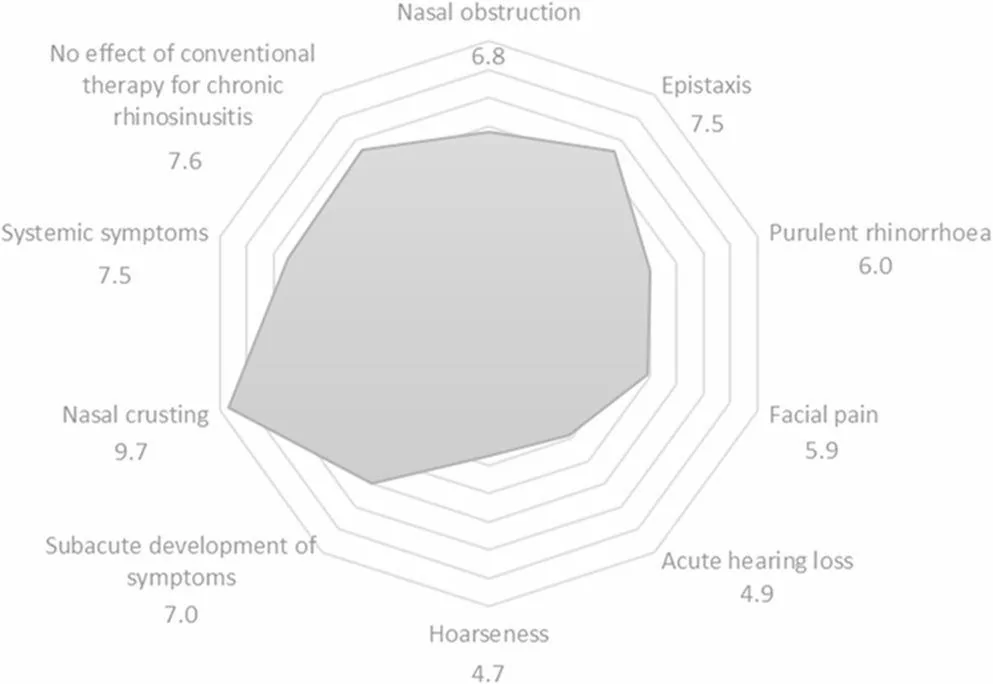
Presenting symptoms and consistency with AAV according to respondents

Otorhinolaryngologists were asked to evaluate and score ten presenting symptoms on their relevance and compatibility with AAV on a 0–10 scale (0 not compatible, 10 very compatible). The highest scoring symptoms were nasal crusting, systemic symptoms, no treatment effect of conventional therapy for chronic rhinosinusitis and/or epistaxis. (Fig. 1).

### Rhinoscopic findings in AAV patients

**Fig. 2:**
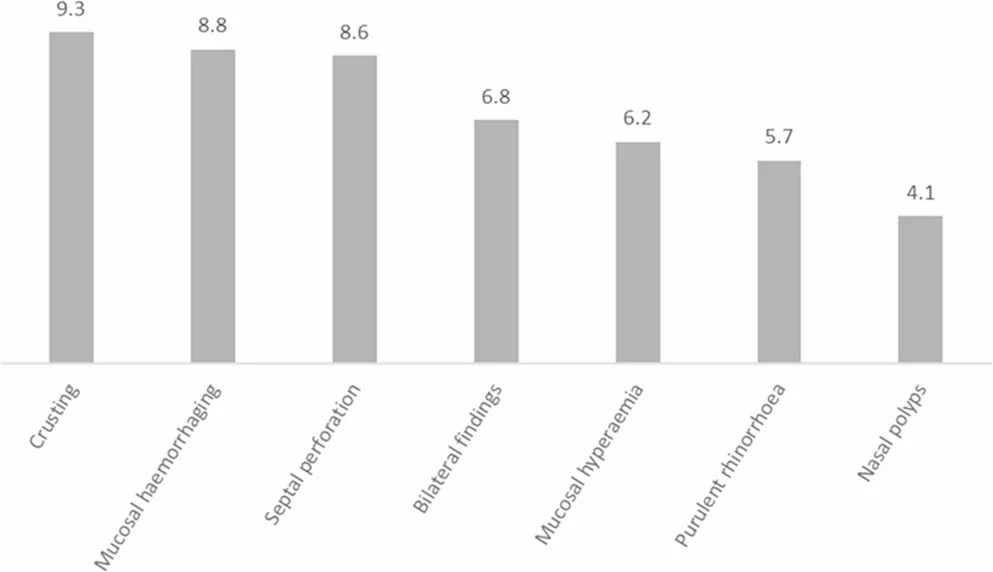
Rhinoscopic findings most suspect for AAV according to respondents

The ENT-specialists were subsequently asked to assess and score seven rhinoscopic findings based on their consistency with AAV on a 0–10 scale. The most suspect finding was nasal crusting followed by mucosal haemorrhaging and septal perforation (Fig. 2).

### Nasal hygiene in AAV

In the in-depth interviews multiple recommendations were formulated concerning nasal hygiene and conservative treatment in ENT involvement. The interviewed otorhinolaryngologists stated that the treatment of sinonasal AAV is particularly challenging due to limited availability of effective local treatment aside from systemic treatment. Furthermore, standard pharmacological treatment alone is often ineffective and surgical interventions can lead to worsening of symptoms. Therefore, nasal hygiene is of great importance. Key recommendations consist of refraining from digital manipulation of the nasal region, frequent high-flow nasal irrigation with a saline solution (minimum of two times per day) and by applying indifferent ointment to the mucous membrane. In doing so, diffuse crusting and potential bacterial overgrowth of the sinonasal cavity is kept at a minimum.

In the statement presented to the respondents regarding nasal hygiene, respondents had a high level of agreement that patients with AAV and ENT involvement should be thoroughly instructed on nasal hygiene (9.1/10).

### ENT follow-up in AAV

To monitor ENT involvement in AAV, the interviewees recommended to perform a rhinoscopy at every follow-up visit, even when patients do not have symptoms. Frequently, rhinoscopy reveals signs of disease activity in patients who present without any sinonasal symptoms. During rhinoscopy there should be special attention for assessing nasal hygiene. In case of suboptimal hygiene, a nasal lavage should be performed prior to inspection of the mucosa. At every consultation, signs of destruction should be recorded for monitoring of progression. Frequency of monitoring visits often ranges from once every two to six months and depends on disease activity and the degree of structural damage. During follow-up, collaboration with a rheumatologist or internal medicine specialist is essential.

Lastly, three statements on the monitoring of ENT involvement were formulated. There was a high level of agreement concerning the statement that ENT involvement should be monitored by an ENT-specialist (8.3/10) and a fair level of agreement (7.6/10) with the statement that nasal lavage should be performed prior to rhinoscopy. Most respondents did not agree with the statement that ANCA titres should be obtained at every consultation with an ENT-specialist (2.6/10).

## Discussion

The current study provides insights into the principal symptoms and rhinoscopic findings that are most compatible with AAV according to Dutch ENT-specialists as well as recommendations regarding treatment and clinical follow-up of AAV. Nasal crusting, poor response to standard therapies, and accompanying systemic symptoms were the primary clinical features that led otorhinolaryngologists to suspect AAV. The most important manifestations of active AAV seen during rhinoscopy were nasal crusting, mucosal haemorrhaging and septal perforation.

In our study ENT-specialists emphasized the importance of universal hygiene recommendations for the ENT region in patients with AAV. Moreover, poor hygiene of the nasal cavity can lead to extensive crusting and/or infection, especially in patients (such as AAV patients) who are receiving immunosuppressive treatment [16]. Systemic treatment of AAV has been widely researched and the EULAR has published recommendations for the management of AAV [17]. However, current recommendations often lack hygiene instructions and local treatment advice. In a review on local management of ENT involvement in AAV, published in 2016, Wojciechowska et al. describe that saline high-flow irrigation of the sinonasal cavity can be beneficial for patients with AAV [18]. Our research underlines this recommendation, adds the recommendations to refrain from digital manipulation of the ENT region, and advises usage of indifferent ointment for the mucous membrane.

To our knowledge, the present study is the first to comprehensively explore and discuss the clinical indicators of ENT involvement, as well as treatment and follow-up recommendations for AAV, from the perspective of otorhinolaryngologists. Identification of AAV activity and distinguishing it from other more common diseases, infections or symptoms due to tissue damage is vital since each clinical presentation warrants a different treatment strategy. In case of persistent AAV activity, changes in the current immunosuppressive therapy should be considered whereas symptoms due to infection benefit from nasal hygiene and/or antibiotics. Currently, no standardized method exists for scoring ENT disease activity in AAV patients and there is no ‘gold standard’ when it comes to follow-up and management of the ENT-region by otorhinolaryngologists. We sought to fill these gaps by investigating which symptoms and rhinoscopic findings can help differentiate between active AAV and other causes such as damage and infections using a standardized, qualitative approach.

Notably, symptom severity was not identified as a factor influencing the consideration of active AAV during the in-depth interviews and was therefore not represented in the extracted themes or incorporated into the subsequent survey. To the authors’ knowledge, there is no literature describing the association between symptom severity and the probability of AAV diagnosis, relative to other conditions with overlapping clinical presentations. Further research on this relationship may clarify whether symptom severity could serve as a useful discriminator between AAV and alternative diagnoses.

A limitation of the study is its explorative nature and the relatively limited number of participants. Due to the explorative nature, no standardized scoring instrument can be made based on the current data.

In addition to the presented data, further research is needed to develop a scoring system helping ENT-specialists to differentiate active AAV from other more common diseases, infections or symptoms due to tissue damage. Such a scoring instrument could not only be used for standardized follow-up and help to better inform treatment decisions in AAV patients but could also be used for standardized evaluation of the ENT-region in future research. Therefore, we aim to perform a follow-up study in which recorded rhinoscopies will be evaluated and scored using a systematic scoring method including the most important characteristics of active AAV as presented in this study to evaluate the accuracy and feasibility of such a scoring method.

In conclusion, key indicators of AAV-activity were nasal crusting, lack of response to conventional treatment and systemic symptoms. The principal rhinoscopic manifestations of active AAV were nasal crusting, mucosal haemorrhaging and septal perforation. These findings could support the development of a standardized scoring system to optimize the diagnostic process and follow-up for AAV.

## Supplementary Information

Below is the link to the electronic supplementary material.

Supplementary Material 1 (DOCX 22.8 KB)

## References

[1] Jennette JC, Falk RJ (2014) Pathogenesis of antineutrophil cytoplasmic autoantibody-mediated disease. Nat Rev Rheumatol 10(8):463–473 10.1038/nrrheum.2014.103 25003769

[2] Jennette JC (2013) Overview of the 2012 revised International Chapel Hill Consensus Conference nomenclature of vasculitides. Clin Exp Nephrol 17(5):603–606 10.1007/s10157-013-0869-6 PMC4029362 24072416

[3] Kitching AR, Anders H-J, Basu N, Brouwer E, Gordon J, Jayne DR et al (2020) ANCA-associated vasculitis. Nat Rev Dis Prim 6(1):71 10.1038/s41572-020-0204-y 32855422

[4] Padoan R, Campaniello D, Felicetti M, Cazzador D, Schiavon F (2021) Ear, nose, and throat in ANCA-associated vasculitis: a comprehensive review. Vessel Plus [Internet]. ;5:41. Available from: 10.20517/2574-1209.2021.41

[5] Felicetti M, Cazzador D, Padoan R, Pendolino AL, Faccioli C, Nardello E et al (2018) Ear, nose and throat involvement in granulomatosis with polyangiitis: how it presents and how it determines disease severity and long-term outcomes. Clin Rheumatol 37(4):1075–1083 10.1007/s10067-018-4019-0 29460094

[6] Comarmond C, Pagnoux C, Khellaf M, Cordier J-F, Hamidou M, Viallard J-F et al (2013) Eosinophilic granulomatosis with polyangiitis (Churg-Strauss): clinical characteristics and long-term followup of the 383 patients enrolled in the French Vasculitis Study Group cohort. Arthritis Rheum 65(1):270–281 10.1002/art.37721 23044708

[7] Durel C-A, Berthiller J, Caboni S, Jayne D, Ninet J, Hot A (2016) Long-Term Followup of a Multicenter Cohort of 101 Patients With Eosinophilic Granulomatosis With Polyangiitis (Churg-Strauss). Arthritis Care Res (Hoboken) 68(3):374–387 10.1002/acr.22686 26315340

[8] Millet A, Pederzoli-Ribeil M, Guillevin L, Witko-Sarsat V, Mouthon L (2013) Antineutrophil cytoplasmic antibody-associated vasculitides: is it time to split up the group? Ann Rheum Dis 72(8):1273–1279 10.1136/annrheumdis-2013-203255 23606701

[9] Srouji IA, Andrews P, Edwards C, Lund VJ (2007) Patterns of presentation and diagnosis of patients with Wegener’s granulomatosis: ENT aspects. J Laryngol Otol 121(7):653–658 10.1017/S0022215106005032 17140461

[10] Dirikgil E, Tas SW, Verburgh CA, Soonawala D, Hak AE, Remmelts HHF et al (2022) Identifying relevant determinants of in-hospital time to diagnosis for ANCA-associated vasculitis patients. Rheumatol Adv Pract 6(2):rkac045 10.1093/rap/rkac045 PMC9245319 35784016

[11] D’Cruz DP, Barnes NC, Lockwood CM (1999) Difficult asthma or Churg-Strauss syndrome? Vol. 318, BMJ (Clinical research ed.). England; pp. 475–6 10.1136/bmj.318.7182.475 PMC1114944 10024248

[12] Corbin J, Strauss A Strategies for Qualitative Data Analysis BT - Basics of Qualitative Research (3rd ed.): Techniques and Procedures for Developing Grounded Theory

[13] Swanson-Kauffman K (1988) E <>Schonwald Phenomenology. NLN Publ 15-2233 97–105 3231529

[14] Dierckx de Casterlé B, Gastmans C, Bryon E, Denier Y (2012) QUAGOL: a guide for qualitative data analysis. Int J Nurs Stud 49(3):360–371 10.1016/j.ijnurstu.2011.09.012 21996649

[15] Tong A, Sainsbury P, Craig J (2007) Consolidated criteria for reporting qualitative research (COREQ): a 32-item checklist for interviews and focus groups. Int J Qual Heal care J Int Soc Qual Heal Care 19(6):349–357 10.1093/intqhc/mzm042 17872937

[16] Lipschitz N, Yakirevitch A, Sagiv D, Migirov L, Talmi YP, Wolf M et al (2017) Nasal vestibulitis: etiology, risk factors, and clinical characteristics: A retrospective study of 118 cases. Diagn Microbiol Infect Dis 89(2):131–134 10.1016/j.diagmicrobio.2017.06.007 28780999

[17] Hellmich B, Sanchez-Alamo B, Schirmer JH, Berti A, Blockmans D, Cid MC et al (2024) EULAR recommendations for the management of ANCA-associated vasculitis: 2022 update. Ann Rheum Dis [Internet]. ;83(1):30 LP – 47. Available from: http://ard.bmj.com/content/83/1/30.abstract 10.1136/ard-2022-223764 36927642

[18] Wojciechowska J, Krajewski W, Krajewski P, Krecicki T (2016) Granulomatosis With Polyangiitis in Otolaryngologist Practice: A Review of Current Knowledge. Clin Exp Otorhinolaryngol 9(1):8–13 10.21053/ceo.2016.9.1.8 PMC4792240 26976020

