# Supplementary material for: Recognition of ear, nose and throat involvement in patients with ANCA-associated vasculitis: from in-depth interviews to insights for the future
Source: Eur Arch Otorhinolaryngol. 2026 Aug 7;283(11):7209–14. doi: 10.1007/s00405-026-10517-0 (PMC13748304; doi:10.1007/s00405-026-10517-0)
Supplement: Supplementary file 1 [file 405_2026_10517_MOESM1_ESM.docx]

**Appendix**

**Appendix I. Interview Guide**

**The interviewer introduces themselves and asks for permission to record the audio. Next the interviewer introduces the subject and the research objectives. If there are no further questions, the interview continues with the following questions.**

**What is the estimated number of (ANCA) vasculitis patients you see per year (for diagnosis/treatment/follow‑up)?**

1. **Recognition of and perspective on AAV in ENT, future prospects**
   a. What are the difficulties in recognizing vasculitis? (in ENT limited AAV)
      i. What are the main differential-diagnostic considerations
      ii. Are there (medical) pitfalls?
   b. Is there a separate component in ENT residency program regarding (ANCA) vasculitis?
   c. What are the bottlenecks for the average ENT specialist (or GP), and how can these be addressed/improved? (e.g. continuing education, courses, publications/presentations at national conferences, incorporation into residency program)
   d. What should future research focus on? (awareness and recognition, measuring disease activity, treatment options?)
   e. How could more awareness for this condition be realized?
2. **Disease activity vs damage**
   a. Which manifestations/presentations/symptoms do you consider most suggestive of vasculitis? (crusts/bleeding/cartilage destruction/polyps/(subglottic) stenosis, etc.)
   b. If the diagnosis vasculitis is being considered, which characteristics/presentations/lifestyle factors should definitely be identified?
   c. Are there features of disease activity that are unique to vasculitis?
   d. How can presence and absence of infection be distinguished?
      i. Disease activity vs. damage?
      ii. Reversible damage vs. irreversible damage?
   e. What advice do you give regarding nose/ear/throat hygiene?
   f. Are adequate local therapies available?
   g. Do you ask about systemic symptoms? (how often are they present?)
   h. What are the most important parameters during follow-up? (medical history/physical exam/biochemical markers/macroscopic manifestations?)
   i. Can patients themselves distinguish between an AAV flare and another cause of symptoms? If so, how?
3. **Examination of the ENT area**
   a. Which instruments do you prefer for physical examination? (rigid/flexible scope)
   b. Do you follow a specific system or methodology during physical examination?
   c. Is there a role for imaging in the evaluation of a patient? (CT/MRI). If yes, when?
      i. What is the additional value of imaging? (diagnostically? assessing response to treatment?)
   d. Is biopsy always necessary for a definitive diagnosis?

**Appendix II. Survey**

Dear colleague,

You are invited to participate in our survey titled 'ANCA-vasculitis in the ENT region'. This survey will take approximately 5–10 minutes to complete. It has been developed in collaboration with the Vasculitis Team at Meander Medical Centre.

Through this survey, we aim to gain insight into how ENT specialists assess, diagnose, and treat vasculitis patients. Your participation is entirely voluntary. All data will be fully anonymized and cannot be traced back to the individual completing the survey.

If you have any questions about the survey or the study, please contact J. Christiaans via email: j.christiaans@amsterdamumc.nl

Your opinion and expertise are highly valuable to us. There are no right or wrong answers in this survey. We appreciate your time and participation. The survey must be completed within two weeks of receiving this invitation.

You can begin the survey by clicking the 'Start' button.

Personal Information

1. I am:
   - ENT resident
   - ENT specialist
2. I currently work in:
   - Regional hospital
   - University hospital
3. I have been an ENT specialist for:
   - 0–5 years
   - 5–10 years
   - Over 10 years
4. How many vasculitis patients have you seen to date?
   - 0–10
   - 10–20
   - More than 20

Assessment of Symptoms

Please rate on a scale from 0 to 10 how characteristic each of the following symptoms is for vasculitis:

| **Symptom** | **Rating (0–10)** |
| --- | --- |
| Nasal obstruction | [ ] |
| Epistaxis | [ ] |
| Purulent rhinorrhoea | [ ] |
| Facial pain | [ ] |
| Acute hearing loss | [ ] |
| Hoarseness | [ ] |
| Subacute development of symptoms | [ ] |
| Nasal crusting | [ ] |
| Systemic symptoms (e.g., general malaise, muscle and joint pain) | [ ] |
| No effect of conventional therapy for chronic rhinosinusitis | [ ] |

Assessment of Rhinoscopy Findings

Please rate on a scale from 0 to 10 how suspicious each of the following findings is for vasculitis during rhinoscopy:

| **Finding** | **Rating (0–10)** |
| --- | --- |
| Crusting | [ ] |
| Purulent rhinorrhoea | [ ] |
| Mucosal haemorrhaging | [ ] |
| Septal perforation | [ ] |
| Mucosal hyperaemia | [ ] |
| Nasal polyps | [ ] |
| Bilateral findings | [ ] |

Recognition and Diagnosis of Vasculitis in the ENT region

Please indicate your level of agreement with the following statements on a scale from 0 to 10:

| **Statement** | **Rating (0–10)** |
| --- | --- |
| Vasculitis is characterised by a chronic course of rhinosinusitis and failure of conservative therapy. | [ ] |
| Patients with vasculitis in the ENT region often complain of facial pain in combination with systemic symptoms (muscle and joint pain, fatigue, general malaise). | [ ] |
| The rhinoscopic image is often characterised by the bilateral occurrence of one or more of the following: crusts, easily bleeding mucosa, destruction of structures including septal perforation, polyposis, subglottic stenosis. | [ ] |
| Almost all patients with active vasculitis have concomitant local infections of the mucosa. | [ ] |
| The differential diagnosis should include cocaine abuse, sarcoidosis, lymphoma. | [ ] |
| Radiological imaging is primarily performed to exclude other causes of inflammation/destruction. | [ ] |
| If there is doubt about the diagnosis of vasculitis, it is recommended to perform a biopsy. | [ ] |
| Even with negative biopsy results, vasculitis may still be present, and treatment may be initiated (biopsies are often false-negative). | [ ] |

Follow-up by the ENT Specialist

Please indicate your level of agreement with the following statements on a scale from 0 to 10:

| **Statement** | **Rating (0–10)** |
| --- | --- |
| Before the mucosa can be properly inspected, a nasal lavage should be performed. | [ ] |
| Patients with vasculitis should be extensively instructed regarding nasal hygiene (such as rinsing, applying ointments, and avoiding digital manipulation). | [ ] |
| Vasculitis in the ENT region is a chronic disease; patients should remain under the care of the ENT specialist (frequency depending on disease burden). | [ ] |
| At each follow-up appointment, ANCA titres should be repeated. | [ ] |

Future Focus

In the future, more attention should be given to (multiple answers possible):

- Creating more awareness for vasculitis in the ENT region and its consequences
- Improving local therapies
- Standardizing hygiene advice for patients
- Education/continuing education for ENT specialists (in training)
- Improving multidisciplinary collaboration

If you would like to add anything or provide further comments, please use the space below:

[Open text field]
